# The fundamental need for unifying phenotypes in sudden unexpected pediatric deaths

**DOI:** 10.3389/fmed.2023.1166188

**Published:** 2023-06-02

**Authors:** Monica H. Wojcik, Annapurna H. Poduri, Ingrid A. Holm, Calum A. MacRae, Richard D. Goldstein

**Affiliations:** ^1^Robert’s Program for Sudden Unexpected Death in Pediatrics, Boston Children’s Hospital, Boston, MA, United States; ^2^Division of Newborn Medicine, Department of Pediatrics, Boston Children’s Hospital, Boston, MA, United States; ^3^Division of Genetics and Genomics, Department of Pediatrics, Boston Children’s Hospital, Boston, MA, United States; ^4^Harvard Medical School, Boston, MA, United States; ^5^F.M. Kirby Neurobiology Center, Boston Children's Hospital, Boston, MA, United States; ^6^Epilepsy Genetics Program, Department of Neurology, Boston Children's Hospital and Harvard Medical School, Boston, MA, United States; ^7^Department of Medicine, Brigham and Women's Hospital, Boston, MA, United States; ^8^Division of General Pediatrics, Department of Pediatrics, Boston Children's Hospital, Boston, MA, United States

**Keywords:** SUDC, SUEND, SIDS, SUDEP, genetics, molecular autopsy, sudden unexpected death in infancy (SUDI), sudden cardiac death (SCD)

## Abstract

A definitive, authoritative approach to evaluate the causes of unexpected, and ultimately unexplained, pediatric deaths remains elusive, relegating final conclusions to diagnoses of exclusion in the vast majority of cases. Research into unexplained pediatric deaths has focused primarily on sudden infant deaths (under 1 year of age) and led to the identification of several potential, albeit incompletely understood, contributory factors: nonspecific pathology findings, associations with sleep position and environment that may not be uniformly relevant, and the elucidation of a role for serotonin that is practically difficult to estimate in any individual case. Any assessment of progress in this field must also acknowledge the failure of current approaches to substantially decrease mortality rates in decades. Furthermore, potential commonalities with pediatric deaths across a broader age spectrum have not been widely considered. Recent epilepsy-related observations and genetic findings, identified post-mortem in both infants and children who died suddenly and unexpectedly, suggest a role for more intense and specific phenotyping efforts as well as an expanded role for genetic and genomic evaluation. We therefore present a new approach to reframe the phenotype in sudden unexplained deaths in the pediatric age range, collapsing many distinctions based on arbitrary factors (such as age) that have previously guided research in this area, and discuss its implications for the future of postmortem investigation.

## Introduction

**Figure 1:**
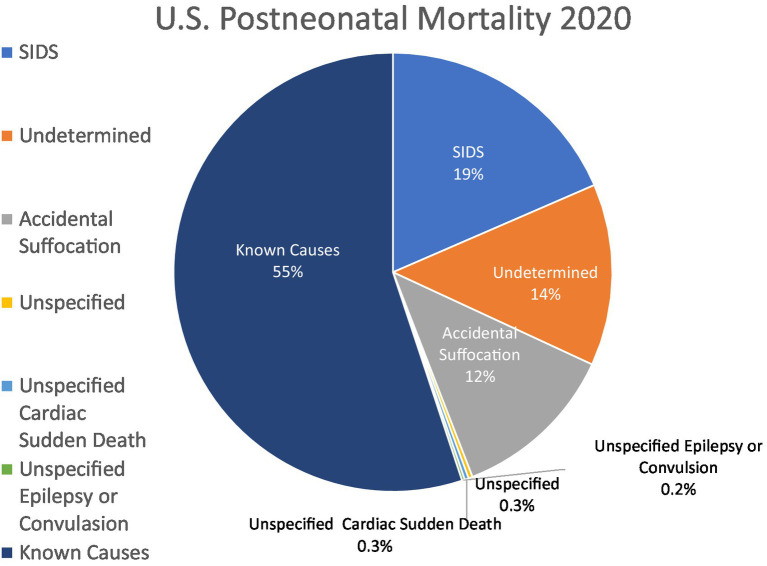
Explained and unexplained US postneonatal mortality, 2020. US postneonatal mortality divided by explained causes of death (55%) and unspecified or other causes of death included in SUID (sudden unexpected infant death; 46%). Data are from Linked Birth/ Infant Deaths Records available through the CDC WONDER database for 2020 (1).

A substantial proportion of infant and early child mortality remains unexplained. When a postmortem examination fails to identify a specific cause for a child’s death, one of a long list of acronyms may be used to certify the death, with terminology emphasizing its sudden, unexpected nature (Table 1). This list includes *sudden unexpected infant death* (SUID), referred to as *sudden unexpected death in infancy* (SUDI) outside the United States (US). SUID and SUDI are intended sometimes to denote a discrete diagnosis used interchangeably with sudden infant death syndrome or “unexplained SUDI,” but at other times they imply a broader category of mortality that includes *sudden infant death syndrome* (SIDS), *undetermined or unclassified cause of death* (Undetermined), and *accidental strangulation or suffocation in bed* (ASSB) (2). SIDS, undetermined, and accidental suffocation are categories used to certify deaths in infants with similar evidence for cause (3), even though the certification of accidental suffocation or positional asphyxia as a cause of death implies objective findings beyond the presence of risk factors (4). When a death without a determined cause occurs in a child over the age of 1 year, the diagnosis of *sudden unexplained death in childhood* (SUDC) may be used, although the term has not been assigned a formal code in the International Classification of Diseases (ICD). Sometimes the cause of death is left *unspecified* and is certified as such. Unexplained (unspecified) *sudden cardiac death or cardiac arrest* and *sudden cardiac death, so described* are other labels for deaths in children that may be unexplained after autopsy, and similarly deaths may be attributed to *epilepsy* or a *convulsion* without a requirement to establish the role or presence of either as a contributor to the lethal event. (Sudden unexplained death in epilepsy, SUDEP, has only recently been given an ICD code.) The above categories comprise 15.9% of deaths in infants and children under the age of 5 years in the United States; between the ages of 1 month and 1 year, they account for nearly half (Figure 1) (1, 5).

**Table 1:** Unexplained US mortality in children 1–10  years, 2020.

	Total mortality	SIDS	Undetermined (other ill-defined and other causes of mortality)	Accidental suffocation	Other sudden death, cause unknown	Unspecified	Unspecified epilepsy/convulsions	Unspecified cardiac arrest
1–27 days	12,879	138	154	87	0	0		0
28–364 days	6,712	1,243	897	817	0	22		20
<1	19,582	1,389	1,062	905	0	22	15	21
1	1,363	0	103	24	32	—	11	—
2	889	0	30	—	—	—	—	—
3	725	0	22	—	—	—	—	—
4	551	0	12	—	—	—	—	—
5	516	0	—	—	—	—	—	—
6	450	0	—	—	—	—	—	—
7	441	0	—	—	—	—	—	—
8	405	0	—	—	—	—	—	—
9	398	0	—	—	—	—	11	—
Suppressed total	144	0	39	9	7	22	50	17
Total	25,320	1,389	1,268	938	39	44	76	38

Annual data for deaths in US children from birth until age 10, focusing on SIDS (ICD R95), Undetermined (ICD R99), Accidental suffocation (ICD W75), Other sudden death (ICD R96), Unspecified (ICD Y34), Convulsions, not elsewhere classified (ICD R56) and Epilepsy, unspecified (ICD G40.9), and sudden cardiac death, so described (ICD I46.1) and cardiac arrest, unspecified (ICD I46.9). Data are from Multiple Cause of Death Files 1999–2020 available through the CDC WONDER Online Database (1). Dashes denote non-zero values with actual numeric value suppressed to prevent identifiability, with an estimated total of 288 deaths.

The nosology of sudden, unexpected pediatric deaths reflects the origins of these categories in the pre-genetic, pre-precision medicine era. When initially proposed in 1969, SIDS included all that would now be considered SUID, SUDI, SIDS, undetermined deaths, ASSB, and SUDC. SIDS, although it contained “infant” in the acronym, was originally defined as *the sudden death of an infant or young child, which is unexpected by history, in which a thorough post-mortem examination fails to demonstrate an adequate cause for death* (6). Essentially, SIDS was meant to label the unexplained death of an infant or child who was not expected to die. The definition of SIDS became restricted to infants under 1 year of age in 1991 as sleep-related factors became implicated (7). Continuing controversies about nomenclature in the field expose fundamental limitations in definitions with little specificity beyond death and age (8). Our program has termed this problem “sudden unexpected death in pediatrics” (SUDP) (9). Hereafter, we will use SUDP as the inclusive umbrella term to incorporate the broader phenomenon of sudden unexpected death in infants and children. We will use SIDS specifically implying the 1991 definition (7), without subtypes (10), and we will use SUID as a term to include SIDS, undetermined deaths and accidental suffocation (unintentional threat to breathing by external cause) under the age of 1 year (11).

## The foundations of SIDS research

A greater understanding of factors that contribute to these deaths has been accomplished through the utilization of pathology and epidemiology in the age-based subgroupings. Current preferences for subcategorization, and the avoidance of the term SIDS as originally proposed, reflect a view that the mortality can be sufficiently explained by identified risk factors including age, sleeping position, and sleep environment in sleep-related deaths with a noncontributory medical history. This preference is chiefly not a consequence of the discovery of new biological markers to establish any specific cause, but rather a result of circumstantial observations and their epidemiologic associations.

### Pathology

SIDS is a condition that presents catastrophically with death and its evaluation involves investigation by pathologists. Since the first published pathological study of 124 SIDS-like infant deaths, investigators have been typically unable to find conclusive evidence for mechanical suffocation based upon autopsy findings (12). Pathological tissue markers for SIDS have been identified: gliosis of the brainstem in areas crucial to respiratory control, increased hepatic erythropoiesis, abnormal thickening of smooth muscle in the walls of small pulmonary arteries, and abnormal relative retention of periadrenal brown fat (13, 14). These markers continue to be documented as nonspecific but recurrent observations on autopsy and may represent surrogate markers for prior sublethal hypoxic exposures. Intrathoracic petechiae in the thymic, cardiac and pulmonary pleura are also commonly noted, suggesting a terminal event that involves negative intrathoracic pressure, yet their presence is unrelated to sleep position (15). However, no pathognomonic features have been elucidated and no biomarker for asphyxia has been developed (16, 17).

It should be noted that, because SUDP involves an unexpected death in a dependent child, a medicolegal autopsy is mandated around the world. In practical terms, mandated autopsies largely compile evidence about the manner of the child’s death to assist in the investigation for possible homicide or neglect and reveal little pertaining to the causes or mechanisms of death beyond trauma, toxicology, and microbiology/virology. In typical practice, these investigations have been insulated from existing and emerging capabilities in molecular autopsy found in academic medical centers (18, 19) and, at least in the United States, the capabilities of the autopsy have changed very little (20). A review of specialized English-language textbooks and sources addressing practice in sudden unexpected deaths in infants and children (4, 21–24) reveals there are no expectations for a molecular autopsy in the routine assessment of SUDP. Unlike emerging standards for the initiation of family studies in early cardiac deaths in adults (25), elements of the molecular autopsy are rarely incorporated in SUDP (26).

### Epidemiology

**Figure 2:**
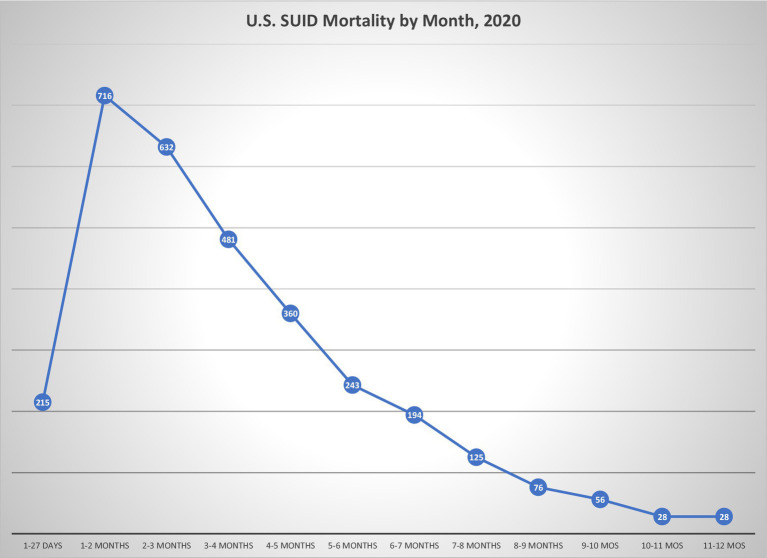
US SUID mortality by age at death. Data of SUID deaths by age of occurrence illustrates relative sparing of youngest neonates, concentrated mortality in ages under 6 months, and continued albeit lesser mortality at older ages. Data are from Linked Birth/ Infant Deaths Records available through the CDC WONDER Online database for 2020 (1).

In contrast, epidemiology directly informs medicine’s approach to SIDS. The initial justification for SIDS as a diagnosis was based on its characteristic pattern of incidence: rare before 1 month of age, peaking between 2 and 4 months, and the major proportion of its mortality occurring before infants reach 6 months of age (Figure 2) (27). This pattern suggests an underlying condition more complicated than developmental vulnerability to airway obstruction and suffocation, since it spares infants whose motor strength and development would be expected to leave them most susceptible to positional asphyxia in the first month of life. A period of concentrated incidence under 6 months is also characteristic of SIDS, and is seen in current data demonstrating that 78.4% of unexplained mortality under the age of 5 years occurred before 6 months of age, followed by continued albeit declining rates of unexplained mortality (5).

Epidemiological research has identified associations between sleep position and environment, and the pathogenesis of SIDS (28, 29). The most commonly cited risk factor for SIDS is prone sleep position [odds ratio (OR):2.3–13.1] (30–32), but soft and shared sleep surfaces are also associated with risk (33). Great credence is given to the fact that SIDS rates dropped by nearly half at a time when placing infants to sleep in the supine position was promoted globally. In some quarters, this is interpreted to mean that infants who die from SIDS are children whose vulnerability is due to sleep environment risks that any normally developing infant would be unable to escape, until they develop the ability to roll at 4–5 months of age. However, the mortality decreases observed in infants during the first years of global campaigns advocating supine infant sleep were not isolated to SIDS mortality alone (34), and similar drops were found even in countries that did not promote supine sleep (35).

### Brainstem serotonin

Unlike efforts to better describe risk for suffocation, most basic science research in SIDS has endeavored to understand how modest risks in the sleep environment may serve as mechanistic triggers for underlying vulnerabilities. Attention to the age of peak incidence in SIDS directed researchers to identify mechanisms to explain data from infants who had died from SIDS while on cardiorespiratory monitors (36, 37). In some of those cases, the observed terminal event was different than what would be expected in suffocation but, instead, involved a failure of “autoresuscitation” characterized by an uncoupling of terminal gasping from the tachycardia that would be expected to accompany it. In the setting of hypoxia and/or hypercarbia, the infants generated gasps after an apneic pause, but the gasps were not accompanied by a cardiac response (38, 39). The infants did not arouse and there was no struggle at the time of death.

Efforts to explain this phenomenon led to landmark research involving the neurotransmitter serotonin in SIDS (40). Serotonin neurons are carbon dioxide sensors that normally cause arousal from sleep and increase ventilation in response to hypercapnia (41, 42). In numerous studies, researchers have shown that levels of serotonin and its binding sites in key brainstem nuclei involved in this response are altered in SIDS (43, 44). Human tissue research has repeatedly shown that the serotonopathy may be present in as many as 40% of infants dying from SIDS (43), and are observed in prone or supine sleep positions and varied environments (45). Subsequently, a mechanism for this auto-resuscitative failure was demonstrated in experimental animal models at a developmental age consistent with SIDS (46, 47). However, in part due to the insulation of SIDS autopsies (as discussed above), serotonin measurements have not translated into standard practice for the postmortem assessment following SIDS, remaining untested in universal forensic practice. The very limited uptake of testing of this pathway makes it of little relevance to clinical medicine, whatever the fatal mechanism involved. Moreover, though serotonin is a factor, little insight has been gained about what causes the serotonopathy.

### Triple risk theory

Taken together, these observations from epidemiology and neuropathology point to a coalescence of factors responsible for death between 1 and 6 months of life, commonly summarized as the “Triple Risk Theory for SIDS” (48). According to the Triple Risk Theory, children who die from SIDS are not “normal” despite having exhibited no overt abnormalities when alive, but they have inapparent underlying vulnerabilities that become fatal when stressed by some external trigger (e.g., hypoxia, hypercarbia, or hyperthermia), and this susceptibility is developmentally or chronologically dependent (48–50). The Triple Risk Theory is commonly held as the working hypothesis for SIDS (33). It may be worth noting that this is the working hypothesis for essentially every disease where the mechanism is unknown.

## Factors challenging the status quo

Despite these advancements, our understanding of the etiology of SIDS is generally recognized to be incomplete. Conclusions from epidemiologic research dominate the current approach, but several factors cannot be adequately addressed by identified risk factors related to sleep position and environment in the pathogenesis of SUID. [Though not discussed here, it should be noted that epidemiology and pathology have both demonstrated the role of cigarette smoking in SUID pathogenesis (51, 52).]

As already stated, SUID spares the youngest infants – even though their motor strength and development would be expected to make them especially vulnerable to positional asphyxia or suffocation risks in the sleep environment. Of the substantial mortality occurring in the first month of life—in 2020, over 75% US mortality in children under 18 years occurred between 1 and 27 days of life—SUID mortality amounted to only 2.9% (1). Although the prioritizing of infant sleep advice is frequently justified by the improved mortality rates that occurred when supine sleep was first promoted in the early 1990s, not all children die in the prone position: recent studies of mortality in the era of supine sleep promotion found over 40% of SIDS were placed (53) or found (54) in the supine position. These studies also detail other risk factors in the sleep environment but other meticulous, population-based research reports 8% of its sample was found supine, alone, on a firm surface, with head uncovered (55).

**Figure 3:**
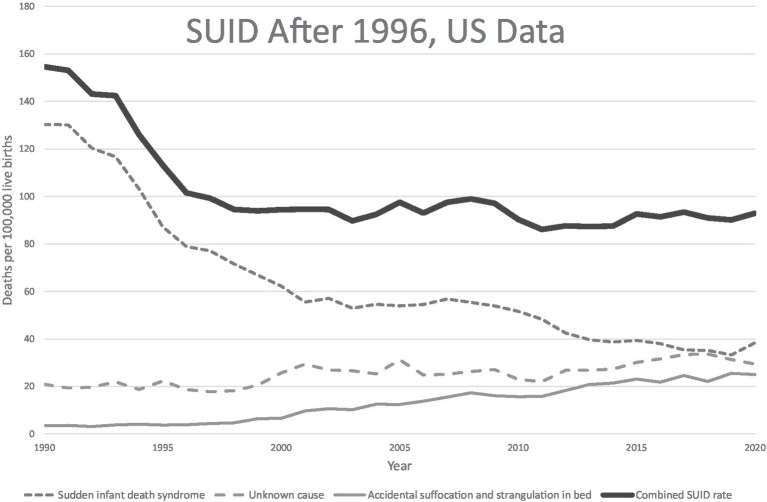
US SUID mortality over the back to sleep era. US mortality rates for SUID and its components in the “Back to Sleep” era. Data from CDC/NCHS, National Vital Statistics System, Mortality Files available at https://www.cdc.gov/sids/data.htm.

Although the focus is typically placed on data showing that only 1–2% of SUID occurs without any risk factors in their sleep environment, the mortality reductions attributed to infant sleep recommendations largely occurred during the initial years of “Back to Sleep,” when only supine sleep position was promoted and before other modifications in the sleep environment became part of the messaging. There has been very limited improvement in overall SUID rates since the inclusion of environmental risk factors in safe sleep campaigns (11, 56), despite three decades of parent guidance about sleep environment (34) (Figure 3). Yet, in 17.7% of SUID cases where suffocation was implicated, it was attributed to soft bedding in 74% (57). Position and sleep environment remain the most important modifiable risk factors, and further improvements are possible with greater adherence to infant sleep advice, but the argument remains that additional strategies are needed to address SUID.

Another gap opened with an explanatory model centered on postural competence is its failure to address deaths over the ages of 4–6 months, when children with normal motor development are able to control their head and neck and have the ability to roll (58). Cohorts in research presenting “risk factors for suffocation” include infants well beyond the age when this developmental vulnerability should be relevant (57). Deaths in infants and children over the age of 6 months with normally-developed postural competence have remained a conceptual wasteland, unaddressed by etiologic hypotheses until older ages in childhood when death from diseases like cardiac arrhythmias or epilepsy are suspected or found (Table 1). There is limited recognition of the size and the significance of this unexplained population, and instead there is a tendency to lump the cause of their deaths into other etiologies. For example, cardiac etiologies are sometimes claimed to be responsible for unexplained deaths in children between 1 and 5 years of age with unrevealing autopsies, even in the vast majority for whom genetic testing fails to identify a gene associated with cardiac disease (59). If these cases parallel the 44% of sudden cardiac deaths without an arrhythmic cause in adults (60), then this should more properly be considered an undiagnosed disease. Similarly, sudden unexplained death in epilepsy, the *sudden, unexpected, witnessed or unwitnessed, non-traumatic, and non-drowning death in patients with epilepsy* (61), requires previously recognized seizures of sufficient complexity to warrant the diagnosis of epilepsy, ignoring the possibility that first seizure events might be lethal. All of this underscores the fact that current practices focus on only some of what are presumed to be many factors in a multifactorial process that leads to death.

Other lines of research have untapped potential in this area. Neuropathologic changes otherwise associated with temporal lobe epilepsy, specifically bilamination in the dentate gyrus of the hippocampus, are found in over 40% of SIDS (62) or SUDC cases (63), in children as young as 6 months up to 16 years (64). Research into SUDEP suggests serotonin mediated mechanisms in common with SIDS and provides new insight into effects on arousal and respiratory control (65). This research suggests that SUDP mortality may occur across a spectrum of diseases with variable ages and severities of presentation, sometimes resembling known disease but at others requiring more than the current approach to diagnosis has to offer.

**Figure 4:**
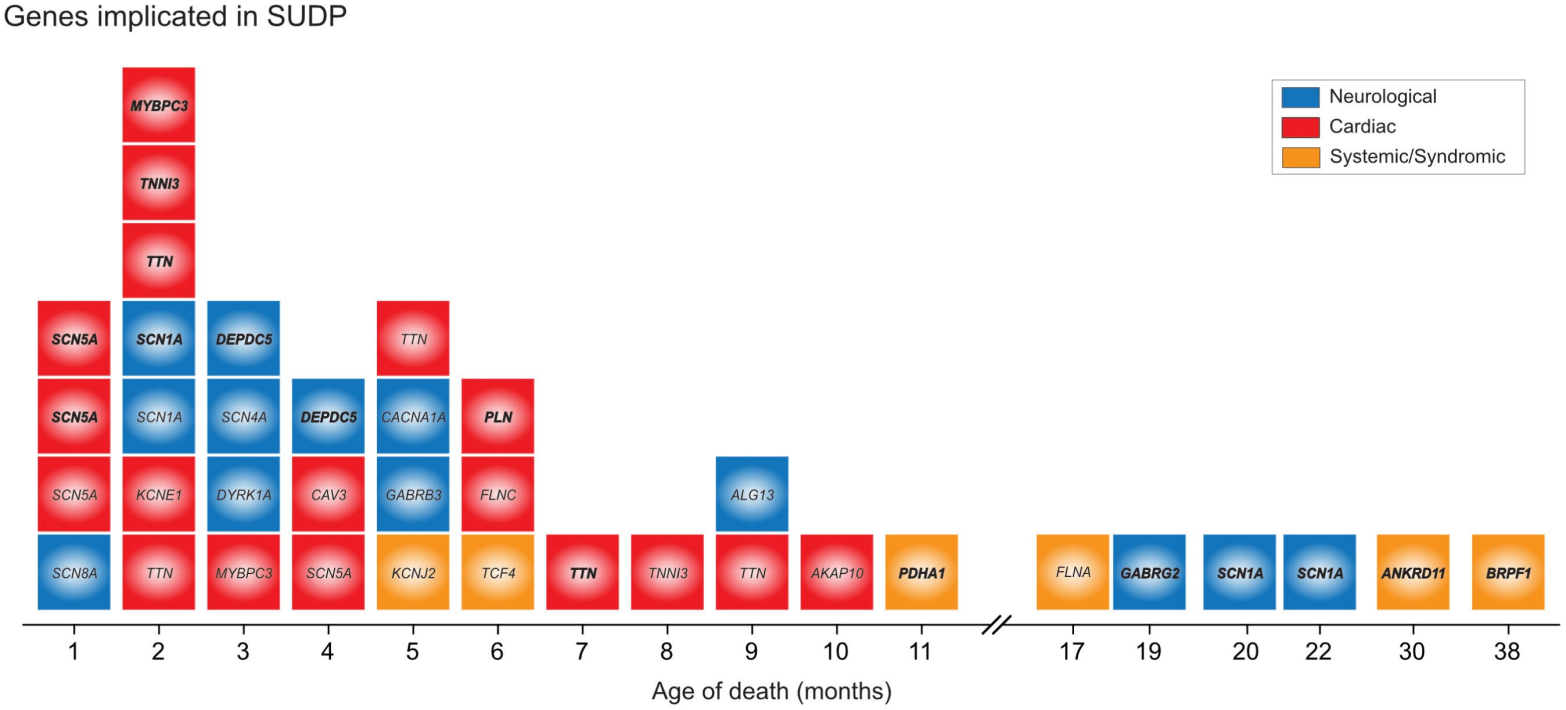
Genetic variants implicated in unexplained deaths in infants and children. Identified pathogenic, likely pathogenic and VUS-leaning pathogenic variants in a cohort illustrate presence of genetic determinant, distribution by age of death mirroring the general epidemiology, and the involvement of common genes that defy the age cut off. By permission, from Koh et al. (73).

Genetic diagnoses found in SUID deaths are often published as case reports (66–70) or nested within larger postmortem (18) or disease-specific (59, 66) cohorts. In recent research involving larger cohorts of SUDP cases, the overall pathogenic genomic variation has become clearer. Potentially causative variants have been identified in metabolic and cardiac genes in 20% of SUID (71) and clinically actionable cardiac variants in 4.3% (72). Our group has reported likely contributory variants in 11% of a well-phenotyped cohort of 352 SUDP cases (73). That work also reported an excess burden of rare damaging variants in genes plausibly linked to sudden death and of rare, *de novo* variants, while others found enrichment in nonsynonymous *de novo* variants in genes associated with cardiac and seizure disorders and a trend to excess loss of function variants in genes intolerant to such variance (74). Still others have found an increased rate of mitochondrial DNA variants in SUDP, the implications of which remain unclear, but could also be related to prior sublethal hypoxic exposures (75). As barriers to population-based genomic sequencing and analysis of these case are slowly overcome, the prevailing age-based categorization and the idea of a unified cause gives way to heterogeneity among infants and children dying from SUDP and analogs in a spectrum of pediatric diseases (Figure 4). Genetic findings defy the unexpected/unexplained categories and challenge the distinctions upon which the categories of SIDS, SUID and SUDC are based, while also highlighting the limited resolution of the phenotype of SUDP.

Although further investigation into the genotype–phenotype spectrum of SUDP is needed to fully define the breadth and depth of “lethal” pathogenic variation, a large issue that researchers confront is the “phenotype gap,” when deleterious variants are identified in cases where terminal mechanisms are unwitnessed and uncertain. This in contrast to children who, on retrospective review of records in the context of a genetic variant, are found to have multiple abnormalities that had either not prompted medical attention or had not been recognized as a syndrome (68). A review of research in SIDS and long QT syndrome (LQTS) highlights many of the hallmarks of SIDS research conducted without a prevailing phenotype. While LQTS now would be considered a separate cause of death from SIDS, the finding that some SIDS deaths were due to LQTS (76) brought attention to the role of channelopathies and their genetic basis in SIDS. Yet, there has been a reluctance to expand the phenotype from a group of patients living with and surviving disease to now include children who were apparently healthy prior to SUDP, even though disease-associated variants (LQTS) were identified. Recognized, validated adult disease may offer direct links between potential mechanisms and otherwise unexplained death, a conclusion that is supported by growing consensus that variable penetrance is typical in most genetic disorders (77).

This problem goes beyond arrhythmias and also includes cardiomyopathy-associated variants in SUDP. Cardiomyopathy is complicated to diagnose in its early stages, requiring the presentation of symptoms at critical points in its progression. It is virtually unexplored in the pre-diagnostic ages before apparent illness, and extreme first presentations are rarely considered. Yet, clinically actionable cardiomyopathy associated variants made up 70% those found in autopsy-inconclusive sudden cardiac deaths (78). The same might be said about epilepsy associated genes, where deleterious variants in *SCN1A* have been reported in Dravet’s syndrome, genetic epilepsy with febrile seizures plus (GEFS+), SIDS, and SUDC (69, 70, 73). Since investigation into genetic causes in these cases is not the current standard of practice, it seems reasonable to expect that there are important insights to be gained. One might imagine a panoply of other disorders contributing to SUDP, each recognizable in their fully penetrant forms yet obscurely uniform in early manifestations associated with sudden death.

It is important to note that, while the disease processes may be stochastic and relatively age-agnostic, pediatric disease and genomic risk factors are not unaffected by medical intervention. The true ‘natural history’ of genetic conditions is impossible to determine in the pediatric age range, as survival is highly dependent upon medical intervention. For example, a sudden cardiac and respiratory collapse in an infant previously thought to be healthy may result in death in one clinical context, whereas the same child presenting to another hospital might be resuscitated, undergo a genetic evaluation, and be found to have a chromosomal deletion disorder causing hypotonia and explaining the unexpected collapse; this same child may then survive into childhood and beyond. Indeed, the population of infants who are chronically critically ill, i.e., reliant on technology with life-limiting impairments, may have in different contexts died and been classified as SIDS/SUDC deaths. Further compounding these challenges is the inability to accurately account for deaths attributable to genetic conditions in mortality statistics, with our recent work demonstrating that a substantial proportion of infant mortality confirmed to be due to a genetic disorder is not reflected as such in mortality statistics (79).

Moreover, the current state of clinical genetics and genomics struggles to identify novel genetic conditions with highly negative selection: genes with such a low negative selection pressure (fitness of zero) that they are not known to have a phenotype other than death, as highlighted in a recent genomic investigation of stillbirth that identified variants unique to perinatal lethal populations (80). The challenge thus lies in identifying these genes, when standard postmortem practice rarely includes exome or genome sequencing and at best includes “sudden death panels” that were developed based upon discoveries in individuals surviving long enough to receive the more expansive genetic assessments in modern clinical medicine. Current knowledge regarding lethal genes and phenotypes is flawed and incomplete.

From an undiagnosed diseases perspective, SIDS represents a reservoir of mortality that can be, in some cases, unaffected or only circumstantially affected by extrinsic risk factors. We must also consider evidence that the mechanisms of sudden unexpected deaths may be diverse and not conform to strict categories of organ-based diseases. Children with sudden cardiac death have double the incidence of antecedent febrile seizures than controls (81); personal or family histories of febrile seizures are over-represented in cohorts of children dying from SUDC (82). Second-trimester maternal serum alpha-fetoprotein levels are directly associated with the risk of SIDS, further suggesting unclear mechanisms (83). A focus on mechanisms that seem straightforward in a critical period before rolling in the Triple Risk may miss important aspects of an infant or child’s susceptibility. The current classifications used in unexplained infant deaths have been crucial in establishing recognition for the problem but a continued focus on a phenotype as generic as death considered with another factor providing relatively limited information such as age, has created a syndrome with a false sense of diagnostic completeness that hinders the pursuit of genetic mechanisms while also asserting a bias against the importance of stochastic processes.

## A new hypothesis

**Figure 5:**
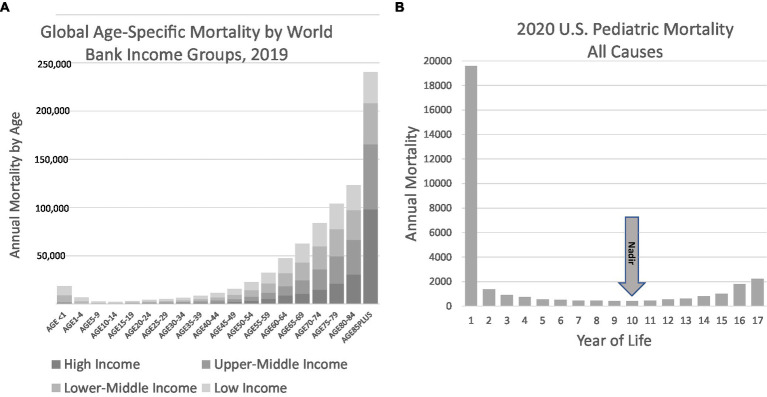
Global mortality and US pediatric mortality by age. **(A)** Global mortality data illustrate the convex nature of mortality, with decreasing mortality in the first decade followed by increases typically attributed to maturity effect. Data obtained from the World Health Organization Global Health Observatory, Life tables (84). **(B)** US Pediatric data illustrates the nadir in mortality to occur in the 10th year of life. Data from the CDC WONDER database for 2020 (4).

We propose a new phenotyping strategy to inform future investigation, particularly from birth until the age of 10 years. Our hypothesis is that sudden unexplained deaths in the pediatric age range are the consequence of undiagnosed diseases and their elucidation is amenable to the techniques of precision medicine. The core population comprising this phenotype is demarcated by human survival statistics that distinguish pediatric mortality trends from mortality rates found later in life (Figures 5A,B). The hypothesis underlying this phenotyping approach and its potential utility are supported by discoveries that have resulted in reproducible biomarkers, albeit in restricted subsets of SUDP cases. We believe that any reframing should build on existing insights and observations and uniformly deploy the best modern medical tools to achieve mortality reductions. These tools include important advances in the molecular or genomic autopsy, the uniform inclusion of family history into basic considerations about disease definition, and a deep and systematic evaluation of the family and its environment. This will bring the diagnostic approach to these deaths into alignment with the strengths of precision medicine. The intent of this phenotyping strategy is not merely for classification and population statistics but, rather, for individual case determination and disease discovery.

The proposed phenotyping strategy incorporates all of the diagnostic categories for sudden unexpected pediatric deaths detailed in Table 1. The categories listed in Table 1 are considered false distinctions by implication, except in cases where evidence for the stated cause can be objectively demonstrated and measured. Accidental suffocation, we should mention, is not entirely excluded as a potential cause of death but, instead, is to be regarded as a specific cause of death that requires objective findings at the death scene or on autopsy, and not merely inference from risks in the sleep environment and position. Sudden cardiac death is not excluded as a cause of death, but its diagnosis must be based on genetic, autopsy, or family findings that support its designation.

A phenotype that systematically incorporates all unexplained early child mortality represents a heterogeneous but distinct population of children dying due to (1) etiologies incompatible with survival presenting catastrophically, (2) mechanisms not typically seen in later life or, if seen, manifesting differently in infancy than adulthood, and (3) mechanisms identical to those observed in adult disorders but obscured in pediatrics by incomplete penetrance, variable expressivity or stochastic occurrence over time. It includes presentations of known diseases not routinely ascertained by current postmortem evaluations, expansion of the phenotypes of known diseases, and novel pathological mechanisms revealed by genomic analysis. The diagnostic potential is seen not only in our work (85, 86), but that of others (87). As a working hypothesis, this category exists on a continuum comprising deaths that occur in the setting of stillbirth, congenital malformations, prematurity, SIDS, SUDC, SUDEP and Sudden Cardiac Death. It approximates recognized, validated disease in some case (more frequently in later childhood) when it presents similarly to SUDEP and sudden cardiac death. Age-based distinctions are not without basis but are not central to the definition of this phenotyping approach.

Instead, this phenotypic umbrella integrates multiple noncontiguous endophenotypes. Endophenotypes are biomarkers that are biologically correlated to an outcome of interest or disease vulnerability, and can be measured in all individuals, whether affected or unaffected. They offer the practical advantage of providing greater power to identify disease-related biomarkers and genes than binary disease categories, especially when compared to the vague phenotype of SIDS (84, 88). Endophenotypes serve as intermediaries between genetic risk and ultimate outcome, and gain definition through their combination of multiple axes of objective data. For example, biomarkers of epilepsy and cardiomyopathy can be found on autopsy or in extended family studies, serotonin binding can be quantified through immunohistochemical assays, or personal and family histories of febrile seizures can be identified in medical histories. Once biomarkers are identified, they provide an enhanced method to reveal the genetic architecture of heterogeneous process under the common umbrella of SUDP. The greater challenge is understanding the patterns of disease these structural features represent.

It will be crucial to increase our understanding of heritable risk within affected families and include it into some endophenotypes. In this regard, the lingering influence of “Meadow’s Law” (89) impedes the discovery of genetic diseases. Familial recurrence studies generate odds ratios of 4.84 for SIDS and other or unknown deaths to sibs at any age or an odds ratio of 9.29 (CI 2.62–32.96) in first through third degree relatives (90). Effects of this magnitude are typically seen with Mendelian disorders or conditions with directly causal environmental influences. Discrete endophenotypes might be revealed through a family history of stillbirth, epilepsy, febrile seizures, sleep disorders, or early cardiac death. Similarly, given that preterm infants are at three-to fourfold higher risk for SIDS than term infants, and the gestational age of peak vulnerability for SIDS appears to occur 4 to 6 weeks earlier among preterm than term infants, it may be productive to investigate SUDP deaths when there were siblings with prematurity. A family history of congenital malformations could be considered an endophenotype. Siblings of children who died and were found to harbor serotonin abnormalities might be another. The study of these endophenotypes will require large, carefully detailed cohorts and collaboration.

The rigorous study of a problem with evidence of heritable contributions typically requires a formal kin-cohort study (91), where family members are phenotyped irrespective of their presumed affected status (likely to be assumed non-affected if alive and over 1 year of age) and based simply on relationship to the proband. The current conundrum in SIDS research stems from the forced uniformity of the available dataset (i.e., sudden death with no evident cause), and so cannot be assumed to reflect any particular subset of conditions or mechanisms (genetic or environmental). As an alternative, any systematic approach would ideally require a consistent minimal dataset not conditioned on the mode of presentation. This minimal dataset should include a standard post-mortem sample collection for genomics, metagenomics, proteomics (92), and environmental toxicology. Neurohormonal, metabolic, or ischemia-related biomarkers would also be of broad utility in testing extant hypotheses for SIDS. Whole body post-mortem MRI has the potential to add a substantive and unbiased dataset to the typical forensic autopsy (93, 94), and here too standard tissue sampling protocols for central digital pathology using modern analytics would minimize the subjectivity in these relatively infrequent evaluations. Family members offer the potential for rigorous assessment of multiple anatomic and physiologic endophenotypes in individuals with a high pretest probability of shared abnormalities, including core cardiac and neurological functional evaluations, or sleep studies that include targeted assessment of physiological responses to hypoxia, hypercarbia, or other external perturbations. Rigorous kin-cohort approaches would mandate the study of at least two generations and would include formal transmission probabilities. Segregation analysis also lends remarkable statistical power to this approach and lays the groundwork for formal molecular genetics. The development of functional genomic biomarkers for asphyxia or suffocation would have tremendous utility not only in pediatrics and several candidates already exist including the induction of hypoxia pathways. Such assays, for example RNA sequencing, may also have the advantage of integrating events over the hours prior to the demise of the infant.

Efficient assessment of core endophenotypes across multiple sites would rapidly define whether any potential phenotype is of utility. This necessarily will require investments in collaborations with medical examiner systems, as these efforts might also be rigorously integrated into forensic autopsies accelerating closure in this harrowing process. They will require referral networks with diverse participation; they will be facilitated by centers of excellence that concentrate the resources necessary to conduct the work.

## Discussion

**Figure 6:**
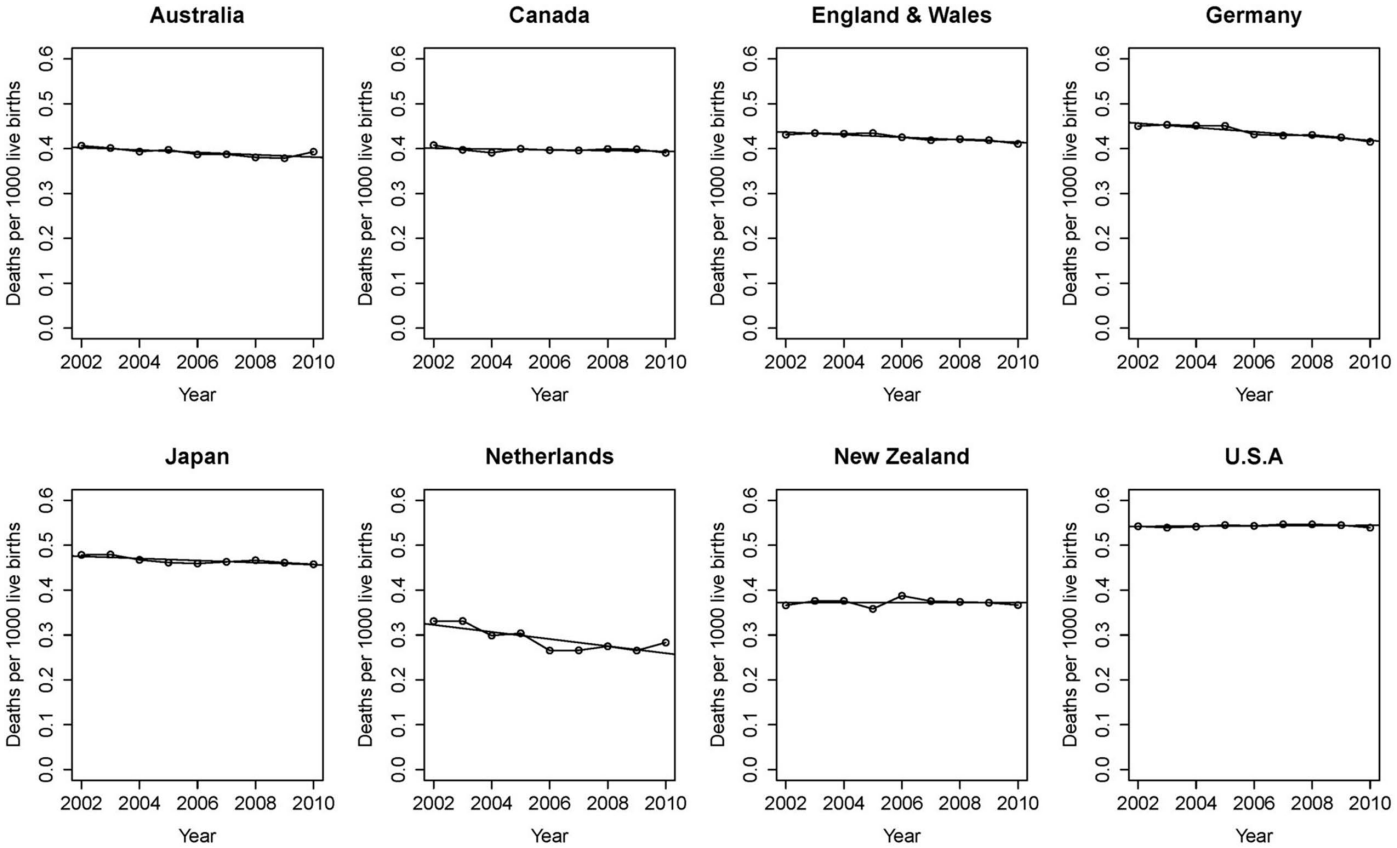
International data shows stalled progress against unexplained mortality. Recommendations for supine, uncluttered, “safer” sleep have been universally embraced for decades. Despite refinements in understanding associated risk in the infant sleep environment, reports from advanced industrial economies fail to demonstrate progress. With permission from Taylor et al. (88).

The 21st century began as the first in recorded history when mortality rates in infants and children are lower than those for adults. This was achieved through advances in public health, hygiene, and nutrition, and by medicine’s steady progress in transforming diseases and their illnesses from mysteries into scientifically understood mechanisms. Some of medicine’s success applies equally in children and adults. The pathogens of infectious diseases are as relevant in pediatrics as they are in internal medicine. But this is not uniformly the case. Childhood cancers, for example, are mostly distinct from those found later in life and their mechanisms differ. As it goes with known disease, so it goes with the unexplained. Burdens of unexplained disease stand out in relief and, while the extant epidemiologic associations dominating the field are valid, the sole focus on them has not demonstrated continuing effectiveness (Figure 6). We propose, instead, to treat sudden unexplained deaths in pediatrics as a broad phenotype of undiagnosed disease conforming to a distinct pattern in human survival. We believe careful phenotyping and genomic evaluation will shed light on individual causes as well as common mechanisms that will only be understood with this focus.

The same factors leading to the proposed hypothesis also motivate a re-evaluation of the Triple Risk Theory. The convergence of individual vulnerability, environment, and child development can no longer be understood as a uniform mechanism or a continuum balancing the relative contributions of intrinsic vulnerability and extrinsic risk (95). There is no *a priori* relationship between extrinsic, intrinsic, and developmental risk. The proposed phenotyping approach encompasses an infant discovered prone and wedged on a couch (endophenotypic feature: circumstances for inescapable suffocation without conclusive biomarker), an infant found supine amidst inconclusive sleep environment risks with lowered serotonin binding in medullary raphé nuclei (endophenotypic feature: serotonin), a toddler with a history of febrile seizures found prone with bilamination of the dentate gyrus (endophenotypic feature: epilepsy *in situ*)*, and* an older child found on autopsy to have an epilepsy-related variant in sodium channels discovered in the prone position (endophenotypic feature: SUDEP without epilepsy). We propose that these deaths are not from the same cause and, moreover, that they present testable hypotheses.

The apparent “mortality gap” reported under 1 month of age also bears scrutiny. The difficult process of labor and delivery followed by an infant’s transition from placenta to independent breathing and circulation—50% of stillbirths occur during labor—may be falsely removed from our considerations. This makes it appear that there is a sparing when in fact the stress of delivery is unlike any challenge an infant or adult will face in their lifetimes and may claim an oversized amount of related mortality. Further complicating the accounting is that once in the NICU, infant deaths are not classified as SIDS, and infants who might otherwise die of SIDS do not. This is not because sudden and unexpected postnatal collapse does not occur; rather, these events either result in survival due to life-saving medical interventions, or in death that is not classified as SIDS. Similarly, possible relationships with preterm deaths and deaths due to congenital anomalies may share commonalities, where deaths occurring in the setting of extreme prematurity or congenital anomalies – which often occur within the first month of life—are not typically thought of or classified as SIDS-related mortality. Indeed, data to understand survival from what otherwise might be considered SIDS is entirely lacking. We point out that dramatic declines in neonatal mortality attributed to advances in neonatal intensive care occurred in parallel with the mortality improvements in SIDS (96).

One additional concept which may be of relevance is that of overall physiologic resilience, where death is a consequence of events that typically would have encountered sufficient homeostatic buffering but in the setting of reduced reserve are unopposed and lethal. This might be imagined to be the mirror image of geriatric vulnerability where the blunting of multiple physiologic responses can lead to unanticipated fatality with apparently minor events or intercurrent illnesses. Importantly, there are some very direct correlates with specific organ systems contributing disproportionately to this reserve. In a pediatric cohort, the failure to build resilience at the relevant rate in these same systems might represent a critical endophenotype, and also is aligned with the observations of SUDP risk in premature infants. What is clear is that current assumptions about the unique attributes of mortality under 10 years leave much to be explored.

This new approach will bring additional metrics for success that go beyond national mortality statistics or parenting practices, if only by incorporating the evaluation of these deaths into clinical medicine, bringing diagnostic and support resources and also accountability to provide an explanation to the child’s family to medicinal practice. Diagnostic yield, the impact of the multidisciplinary evaluation on family health, and the impact on healthcare utilization become newly relevant. Importantly, the uniformity of family assessment may also help destigmatize what is a remarkably challenging investigatory period. The effect of an agreed strategy on the understandable but disproportionate impact of potential infanticide and access to cases may be salutary.

There are several limitations that are unique to genomic analysis and disease discovery in this area. Variant interpretation is a particular challenge, in part due to limited phenotype information. Whereas variants of uncertain significance in living individuals may be resolved through additional clinical investigation or functional evaluation, this is typically not possible for variants identified post-mortem, where samples for functional studies do not exist and it is impossible to know if the decedent would ever develop disease manifestations typically associated with the gene in question. Aside from these phenotyping challenges that limit the clinical interpretation of variants identified, the potential for incomplete penetrance—as seen in genetic cardiomyopathies, cardiac channelopathies, and genetic epilepsy syndromes—presents a barrier to the use of population frequency in attributing variant pathogenicity. For example, multiple individuals in the same family may harbor a pathogenic variant in a cardiomyopathy gene, though the disease manifestation may range from asymptomatic to perinatal lethal. Thus, a traditional approach to Mendelian genomic analysis for early-onset rare disease, where conditions are assumed to be rare and highly penetrant, may not be successful in the majority of cases of SIDS.

A frequently stated concern is the possibility of making postmortem diagnoses we are unable to prevent. There is the additional probability of determining causes for which there is no prevention or treatment. While this reservation can be stated out of concern for families, it must be remembered that in current practice, families after SIDS must make their accommodations to the unknown in the absence of actual facts. If the concerns are for the knowledge base for the sound practice of medicine, the only way to gain information is to do more.

Many diseases remain of unknown cause and, when known, their causes can be complex and involve the interplay of numerous factors, both intrinsic and extrinsic (97). One sobering consequence of the typical approach to SIDS has been to deem it a failure by parents to heed advice. We submit an alternative view: there is a large reservoir of unexplained pediatric mortality that has defied previous efforts by medical science to understand and ameliorate it. Progress will require a different rationale. We therefore introduce a novel approach to phenotyping SUDP, taking into consideration recent genomic advances that have illuminated contributions not previously suspected, while not discounting epidemiologic observations. We believe new objective tools bring us to a threshold for discovery, one promising answers and solace for more families who experience the heartbreaking tragedy of SUDP and must live with evaluations that can seem incomplete. We believe this will provide the critical means to better understand which children are at risk, begin to screen for genetic and/or biological factors, and thus begin a new epoch of prevention.

## Data availability statement

The original contributions presented in the study are included in the article/supplementary material, further inquiries can be directed to the corresponding author.

## Author contributions

MW and RG drafted the work. MW, AP, IH, CM, and RG made substantial contributions to the conception or design of the work, revised the work critically for important intellectual content, provided approval for publication of the content, and agreed to be accountable for all aspects of the work in ensuring that questions related to the accuracy or integrity of any part of the work are appropriately investigated and resolved. All authors contributed to the article and approved the submitted version.

## Funding

MW was supported by K23 HD102589. RG, AP, and IH were supported by the Robert’s Program Fund at Boston Children’s Hospital.

## Conflict of interest

The authors declare that the research was conducted in the absence of any commercial or financial relationships that could be construed as a potential conflict of interest.

## Publisher’s note

All claims expressed in this article are solely those of the authors and do not necessarily represent those of their affiliated organizations, or those of the publisher, the editors and the reviewers. Any product that may be evaluated in this article, or claim that may be made by its manufacturer, is not guaranteed or endorsed by the publisher.

## Author disclaimer

The content is solely the responsibility of the authors and does not necessarily represent the official views of the National Institutes of Health.
